# Evaluation of crAssphages as a potential marker of human viral contamination in environmental water and fresh leafy greens

**DOI:** 10.3389/fmicb.2024.1374568

**Published:** 2024-03-28

**Authors:** Soo Hwan Suh, Jeong Su Lee, Seung Hwan Kim, Jan Vinjé, Soon Han Kim, Geun Woo Park

**Affiliations:** ^1^Division of Microbiology, National Institute of Food and Drug Safety Evaluation, Ministry of Food and Drug Safety, Cheongju-si, Republic of Korea; ^2^Division of Emerging Virus Vector Research, National Institute of Health, Korea Disease Control and Prevention Agency, Cheongju-si, Republic of Korea; ^3^Division of Viral Diseases, National Center for Immunization and Respiratory Diseases, Centers for Disease Control and Prevention, Atlanta, GA, United States

**Keywords:** crAssphage, norovirus, human virus indicator, irrigation water, fresh leafy greens

## Abstract

CrAssphages are human gut bacteriophages with potential use as an indicator of human fecal contamination in water and other environmental systems. We determined the prevalence and abundance of crAssphages in water, food, and fecal samples and compared these estimates with the prevalence of norovirus. Samples were tested using two crAssphage-specific qPCR assays (CPQ056 and TN201-203) and for norovirus using TaqMan realtime RT-PCR. CrAssphage was detected in 40% of human fecal specimens, 61% of irrigation water samples, 58.5% of stream water samples, and 68.5% of fresh leafy greens samples. Interestingly, across all sample categories, crAssphage concentrations were 2–3 log10 higher than norovirus concentrations. The correlation of detection of crAssphage and norovirus was significant for the irrigation water samples (*r* = 0.74, *p* = 7.4e-06). Sequences obtained from crAssphage positive samples from human fecal and stream water samples phylogenetically clustered with genotype I crAssphages, whereas sequences derived from irrigation water samples clustered differently from other genotypes. Our data show that crAssphages were prevalent in norovirus-positive water samples and in fresh leafy green samples, there was a strong correlation between the presence of crAssphage and norovirus. CrAssphage genomic copies were consistently higher than norovirus copies in all sample types. Overall, our findings suggest that crAssphages could be used as reliable indicators to monitor fecal-borne virus contamination within the food safety chain.

## 1 Introduction

Worldwide, human norovirus (HuNoV) is a leading cause of acute gastroenteritis (AGE) and associated with an estimated 684 million cases of AGE resulting in societal costs of approximately USD 60 billion per year (Cates et al., 2020). The primary route for HuNoV transmission is the fecal-oral route, which can occur through person to person contact with infected individuals or by consuming contaminated food or water (Ahmed et al., 2014). Most common sources of foodborne norovirus outbreaks are ready-to-eat foods that contain fresh produce and mollusks that are eaten raw, such as oysters (Morton et al., 2009; Hall et al., 2012; Grove et al., 2015).

Traditionally, fecal indicator bacteria (FIB) such as *Escherichia coli*, are widely employed as a tool to monitor fecal pollution [World Health Organization, 2012, Bacteriological Analytical Manual (BAM) | FDA]. However, the presence of FIB does not indicate that the contamination is human origin or provides information about the presence of human pathogenic viruses such as hepatitis A virus and norovirus (Jennings et al., 2020). Methods to detect these viruses in produce have been described (Li et al., 2018), but cost of testing, longer testing times, and relatively low viral loads present in produce samples are major limitations to implement routine monitoring for such viruses in fresh produce (Sabar et al., 2022).

CrAssphage, one of the most ubiquitous human gut viruses that are not associated with any known disease, has emerged as a promising indicator for human fecal contamination because it is widely prevalent in a human population and not in animal feces from poultry, swine, and cats (Dutilh et al., 2014; Farkas et al., 2018; Sabar et al., 2022). Several studies have reported a higher abundance of crAssphages and their stronger correlations with enteric viruses than other fecal markers, supporting its use as a human specific source tracking marker in sewage and rivers (Stachler et al., 2018; Ballesté et al., 2019; Farkas et al., 2019; Malla et al., 2019; Park et al., 2020; Nam et al., 2022; Mafumo et al., 2023).

In recent years, several PCR assays for the detection of crAssphage have been developed including CPQ056, which targets the ORF00024 region associated with a hypothetical protein in the crAssphage genome (JQ995537) (Stachler et al., 2017). This assay has been used to monitor fecal pollution in water systems or fecal contamination in oysters (Stachler et al., 2018; Ahmed et al., 2019a,b; Farkas et al., 2019; Gyawali et al., 2021). The CDC developed a real-time PCR assay based on the conserved regions of the DNA polymerase gene (ORF00018), utilizing data from 43 publicly available crAssphage sequences (Park et al., 2020). This assay has proven useful for the detection of crAssphage on hard surfaces during norovirus outbreaks on cruise ships and long-term care facilities, as well as in clinical specimens (Park et al., 2020; Cannon et al., 2022). Despite their widespread use, a comparative study evaluating the sensitivity of these approaches has not yet been conducted.

In this study, we investigated the potential of crAssphage as a biomarker for detecting fecal-borne virus contamination and possible foodborne viruses in various water sources and fresh produce within the food safety chain. Specifically, our approach involved quantifying crAssphage concentrations in environmental water samples, including those from rivers and irrigation systems, and in fresh leafy greens. We then compared these concentrations with the presence of HuNoV. To improve the detection rate of crAssphage, we utilized both two distinct crAssphage assays, developed by the US Environmental Protection Agency (EPA) and the US Centers for Disease Control and Prevention (CDC).

## 2 Materials and methods

### 2.1 Sample collection

Human, animal fecal, and environmental water samples were collected and analyzed for the presence of norovirus and crAssphages. Norovirus-positive and healthy human fecal specimens (*n* = 76) collected from 2019 to 2020 were sourced from the Chungnam Public Health and Environment Research Institute (Hongseong-gun, Chungcheongnam-do, South Korea). Archived animal fecal specimens (bovine, *n* = 100) from a previous study conducted from 2017 to 2019 (17162MFDS034) were used to detect crAssphages and norovirus. We collected samples of stream water (*n* = 201) and irrigation water (*n* = 161) from various regions of South Korea. All irrigation water used in the surveyed fields was sourced from groundwater, with each sampled leafy greens having a water tap connection to this source. Fresh leafy greens (*n* = 64, cabbages = 26, lettuces = 38) were collected from the same or similar sites where the irrigation water samples were taken.

### 2.2 Sample processing

Human and animal stool suspensions (10%) were prepared in phosphate-buffered saline (Gibco) and the solids were removed by centrifugation at 3,000 × *g* for 10 min. The supernatant was then collected for nucleic acid extraction.

Stream and irrigation water samples (600 mL) were collected in disposable Whirl-Pak bags from the mid-river at the surface and irrigation water sources. Samples were stored in the dark at 4°C for less than 6 h until processed in the laboratory. Prior to filtration and concentration, samples were centrifuged at 10,000 × *g* for 30 min at 4°C. The supernatant was subsequently filtered through a 0.22 μm pore-size syringe filter (Millipore) followed by concentration through single-use 0.05 μm polysulfone hollow fiber filter tips (InnovaPrep) in conjunction with the CP-Select™ (InnovaPrep). Viral particles collected on the filter tips were eluted in 500 μl of 0.075% Tween-20/25 mM Tris. The eluant was used for nucleic acid extraction. Fresh leafy greens samples (100 gram) were placed in a glass flask containing 950 mL of recovery solution (TGBE; 100 mM Tris-HCl, 50 mM glycine, 3% beef extract, pH 9.5) (Foodborne Pathogen Investigation Test Methods, Ministry of Food and Drug Safety, 2023). After shaking at 150 rpm for 1 h, 500 mL was centrifuged at 6,000 × *g* for 10 min. The supernatant was transferred to a sterile flask, then mixed with 427 mL of 40% polyethylene glycol (PEG) 8000 and 142 mL of 3M NaCl and stirred at 4°C for 16 h. Following centrifugation at 16,000 × *g* for 20 min at 4°C, the supernatant was discarded. The pellet was mixed with 15 mL of diethyl pyrocarbonate (DEPC) water and 20 mL of chloroform:isoamyl alcohol (24:1), then shaken vigorously for 5 min. The mixture was centrifuged again at 10,000 × *g* for 30 min at 4°C and then 9 mL of 40% PEG 8000 and 3 mL of 3M NaCl was added and incubated for 3 h at 4°C. After centrifugation at 35,000 × *g* for 20 min at 4°C, the supernatant was removed and the remaining pellets were resuspended in 3 mL of DEPC water prior to nucleic acid extraction.

Nucleic acids were extracted using the QIAamp Viral RNA Mini QIAcube Kit (Qiagen, Hilden, Germany) and QIAcube (Qiagen) following manufacturer’s instructions. The total extracted nucleic acid (DNA and RNA) concentration was measured using a spectrophotometer (NanoDrop One; Thermo Scientific, Waltham, Massachusetts, USA) and adjusted to a concentration of 20 ng/mL.

### 2.3 Molecular assays

#### 2.3.1 CrAssphage detection

To detect crAssphages, we employed two previously reported real-time qPCR assays, CPQ056 (Stachler et al., 2017) and TN201-203 (Park et al., 2020). Both assays were used to detect crAssphages in leafy greens, environmental water (stream and irrigation water), and fecal samples. The oligonucleotide primers and probes used in the assays are summarized in Table 1. The qPCR assays were performed as previously described (Stachler et al., 2017; Park et al., 2020) on a 7500 Fast Real-Time PCR system (Applied Biosystems, USA). To quantify crAssphages, a 10-fold serially diluted quantified amplicon was used to generate a standard curve. Based on the standard curve and the cut-off of Ct > 40, the Limit of Quantification (LOQ) was estimated as 1.7 × 10^3^ and 2.0 × 10^3^ copies for CPQ056 and TN201-203, respectively.

**TABLE 1:** Primers and probes used in the present study.

Virus	Primer/probe	Sequence (5′→3′)	References
CrAssphage	CPQ056_F	CAGAAGTACAAACTCCTAAAAAACGTAGAG	Stachler et al., 2017
	CPQ056_R	GATGACCAATAAACAAGCCATQTAGC	
	CPQ056_P (Probe)	FAM-AATAACGATTTACGTGATGTAAC-TAMRA	
	TN201	ATGTWGGTARACAATTTCATGTAGAAG	Park et al., 2020
	TN203	TCATCAAGACTATTAATAACDGTNACAACA	
	TN202 (Probe)	FAM-ACCAGCMGCCATTCTACTACGAGHAC-TAMRA	
	JP1crasF	TAAAACTACWATTTATAGAGTTAATAAAGATGCSTTTAGT	
Norovirus GI	COG1F	CGYTGGATGCGNTTYCATGA	Lee et al., 2017
	COG1R	CTTAGACGCCATCATCATTYAC	
	RING1(a)-TP (Probe)	FAM-AGATYGCGATCYCCTGTCCA-TAMRA	
Norovirus GII	BPO-13	AIC CIA TGT TYA GIT GGA TGA G	
	BPO-13N	AGT CAA TGT TTA GGT GGA TGA G	
	BPO-14	TCG ACG CCA TCT TCA TTC ACA	
	BPO-18 (Probe)	VIC-CAC RTG GGA GGG CGA TCG CAA TC-TAMRA	

For sequencing, PCR-positive crAssphage samples were amplified using oligonucleotide primers (JP1crasF/TN203) to generate a 1089-bp PCR amplicon (Table 1). The purified PCR products were sequenced using an ABI Prism 3500 × L genetic analyzer and a BigDye Terminator cycle sequencing mix (Applied Biosystems, Foster City, CA, USA).

#### 2.3.2 Human norovirus

Human norovirus (HuNoV) GI and GII were detected separately using oligonucleotide primers and TaqMan probes with the protocol reported by Lee et al., 2017 (Table 1). Specifically, HuNoV was amplified using a one-step real-time RT-PCR kit (AgPath-ID One step RT-PCR Kit, Thermo Fisher Scientific). Samples were considered negative if their Ct values were > 40. HuNoV GI- and GII-specific standard curves were generated using 10-fold serial dilutions (10^7^–10^1^ copies) of purified norovirus GI or GII cDNA plasmids as described in foodborne pathogen investigation test methods (Ministry of Food and Drug Safety, 2023).

### 2.4 Sequence and phylogenetic analyses

Multiple sequence alignments of the crAssphage sequences were performed using MUSCLE (Edgar, 2004), and maximum-likelihood phylogenetic trees were constructed using MEGA X (ver. 10.0.4) (Kumar et al., 2018). Pairwise sequence alignment and identity calculations were performed using the Sequence Demarcation Tool (SDT v. 1.2) (Muhire, 2014).

### 2.5 Correlation analysis of norovirus and crAssphages

Pearson’s linear correlation analysis was performed between viral concentrations using R (R Core Team, 2022, version 4.13) within RStudio (RStudio Team, 2016, version 2022.07.1), and figures were prepared using the packages ggplot2 (Wickham, 2016) and ggpubr (Kassambara, 2018).

### 2.6 Performance of crAssphage as a marker of norovirus

Marker performance indicators were calculated as follows: sensitivity is defined as the proportion of positive samples in which the marker was detected, and specificity is defined as the proportion of negative samples in which the marker was not detected (Trullols et al., 2004).


$$S\,e\,n\,s\,i\,t\,i\,v\,i\,t\,y=\frac{T\,P}{\left(T\,P+F\,N\right)}$$

$$S\,p\,e\,c\,i\,f\,i\,c\,i\,t\,y=\frac{T\,N}{\left(T\,N+F\,P\right)}$$

True positive (TP) refers to the number of norovirus-positive samples, and false negative (FN) refers to the number of norovirus-positive and crAssphage-negative samples. True negative (TN) refers to the number of norovirus-negative samples, whereas false positive (FP) refers to the number of norovirus-negative and crAssphage-positive samples.

## 3 Results

### 3.1 Prevalence of norovirus and crAssphages

**FIGURE 1:**
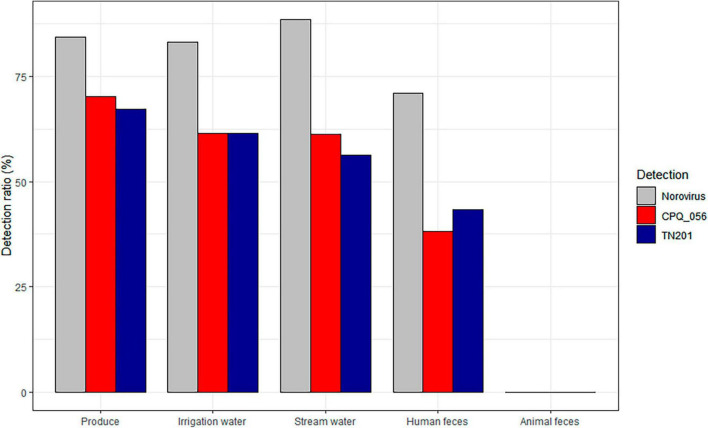
Detection of norovirus and crAssphages in different sample matrices.

**FIGURE 2:**
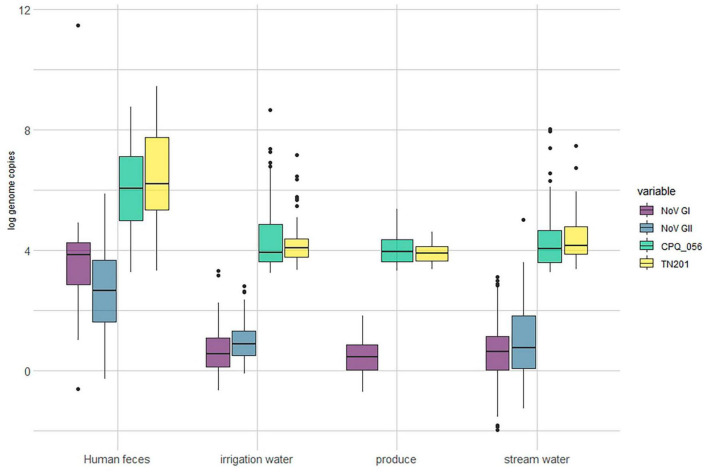
Log concentration (genome copy per gram/mL) of norovirus and crAssphages in samples of produce (leafy greens) (*n* = 64), human feces (*n* = 76), irrigation water (*n* = 161) and stream water (*n* = 201).

Out of the human stool samples tested, 71% (54/76) tested positive for norovirus (either GI or GII). The detection rates for crAssphages in these samples demonstrated a noticeable range between 38 and 43%, depending on the protocol used (CPQ056 vs. TN201-203) (Figure 1). The mean average concentrations of norovirus GI and GII in the human fecal samples were 3.93 (±3.13) and 2.59 (±1.48) log gc/g, respectively, whereas those of crAssphage CPQ056 and TN201-203 were 5.96 (±1.55) and 6.24 (±1.75) log gc/g, respectively (Figure 2).

No norovirus or crAssphages were detected in the animal (bovine) fecal samples (*n* = 100). Norovirus (GI and/or GII) was detected in 83% (134/161) of the irrigation water samples and in 88% (178/201) of stream water samples. Furthermore, crAssphages were detected by both CPQ056 and TN201-203 assays in 61% (99/161) of the irrigation water samples. In the stream water samples, however, there was some variability: CPQ056 protocol detected crAssphages in 61% (123/201) of samples, while TN201-203 protocol showed a slightly lower positivity rate of 56% (113/201). The concentrations of norovirus GI and GII and crAssphage (CPQ056 and TN201-203) in the stream water samples were 0.61 (± 0.92), 0.98 (±1.25), 4.27 (±0.92), and 4.38 (±0.73) log gc/g, respectively, whereas in irrigation water samples the concentrations were 0.66 (±0.72), 1.00 (±0.73), 4.39 (±1.10), and 4.17 (±0.66) log gc/g. Of the leafy green samples, 84% (54/64) tested positive for norovirus GI (no GII detected) and 70% (45/64) and 67% (43/64) samples tested positive for crAssphage using protocols CPQ056 and TN201-203, respectively. The concentration of norovirus GI was 0.49 (±0.60) log gc/g, whereas crAssphage CPQ056 and TN201-203 were detected with concentrations of 4.03 (±0.52) and 3.91 (±0.32) log gc/g, respectively (Figure 2).

There were slight variations in the test results obtained using two PCR assays. The TN 201-203 assay identified a higher number of positives in human fecal samples compared to the CPQ 056 assay (33 vs. 29). Conversely, more leafy green samples tested positive using the CPQ 056 assay than the TN 201-203 assay (45 vs. 43). However, the difference between combined positivity rate and co-positive rate for human fecal samples was 5.2% (43.4 vs. 38.2%). The differences were more pronounced for most of the environmental samples, 80.7 vs. 42.2% for irrigation water, 77.1 vs. 40.3% for stream water, and 81.3 vs. 56.3% for leafy greens demonstrating that the CPQ056 assay more broadly detect crAssphages in environmental samples (Table 3).

**TABLE 2:** Performance indicators for evaluation of crAssphages as a detection marker of human norovirus (GI and GII).

Sources	CPQ056		TN201	
	Sensitivity	Specificity	Sensitivity	Specificity
Leafy greens	0.76	0.56	0.74	0.56
Irrigation water	0.74	0.68	0.74	0.68
Stream water	0.72	0.64	0.69	0.72
Human feces	0.61	0.70	0.62	0.64

**TABLE 3:** Comparison of the CPQ056 and TN201-203, two crAssphage PCR assays, in testing human feces and environmental samples.

Items	# of total samples	Positivity rate (%) by individual assay (# of positive samples)		Combined positive rate (%) by either assay (# of positive sample)	Co-positivity rate (%) by both assays (# of positive samples)
		CPQ056	TN201-203		
Human feces	76	38.2 % (29)	43.4% (33)	46 % (35)	35.5 % (27)
Irrigation water	161	61.5% (99)	61.5% (99)	80.7 % (130)	42.2% (68)
Steam water	64	70.3% (45)	67.2% (43)	81.3 % (52)	56.3% (36)
Leafy greens	201	61.2% (123)	56.2% (113)	77.1 % (155)	40.3% (81)

### 3.2 Correlation between norovirus and crAssphage concentration in different sample matrices

The average slopes of the standard curves were 3.32 (*r*^2^ = 0.995) and 3.79 (*r*^2^ = 0.992) for norovirus GI and GII, and 3.62 (*r*^2^ = 0.997), and 3.54 (*r*^2^ = 0.999) for crAssphage CPQ056 and TN201-203 (Figure 2). Genomic copies of norovirus GI and GII in the environmental water and leafy green samples were 2–3 log10 lower than in the human fecal samples. Interestingly, across all sample categories, crAssphage concentrations were roughly 2–3 log10 higher than norovirus concentrations.

### 3.3 Evaluation of crAssphage as a detection marker of norovirus

**FIGURE 3:**
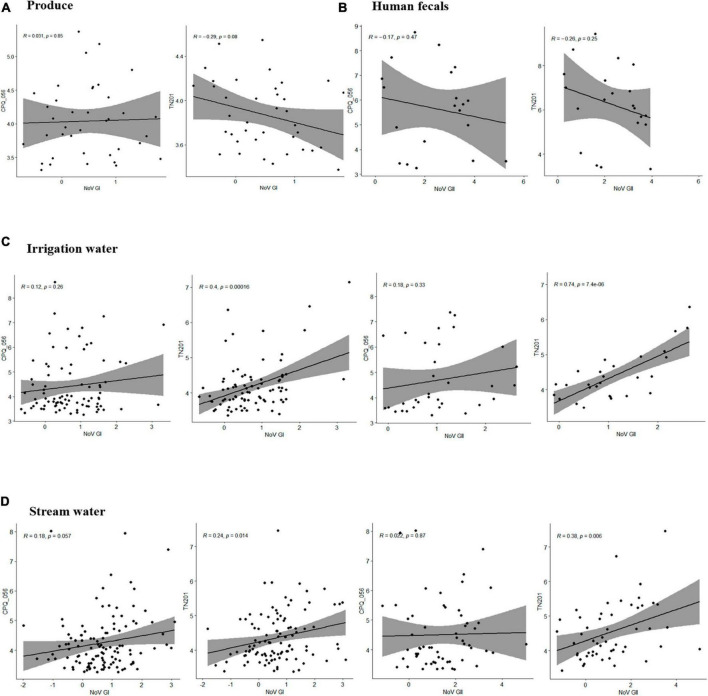
Pearson correlation between human norovirus and crAssphage titers in samples of **(A)** produce (leafy greens), **(B)** human feces, **(C)** irrigation water, and **(D)** stream water. Pearson linear correlation was calculated between viral concentrations using R (version 4.1.3) within RStudio (version 2022.07.1), and figures were prepared using packages ggplot2 and ggpubr.

To determine how crAssphage can function as a marker for norovirus, the sensitivity and specificity were calculated. CrAssphages presented average sensitivity values of 0.75, 0.74, 0.71, and 0.62 in the fresh leafy greens, irrigation water, stream water, and human fecal samples, respectively. In contrast, the average specificity values of the two protocols (CPQ056 and TN201-203) were 0.56, 0.68, 0.68, and 0.67, respectively (Table 2). A weak correlation (*r* < 0.5) was seen among viral titers, except for TN201-203/norovirus GII in irrigation water samples, where the correlation was significant (*r* = 0.74, *p* = 7.4e-06). TN201-203 had a slightly stronger correlation with norovirus than CPQ056 in water samples (Figure 3).

### 3.4 Phylogenetic analysis and association of norovirus and crAssphages

**FIGURE 4:**
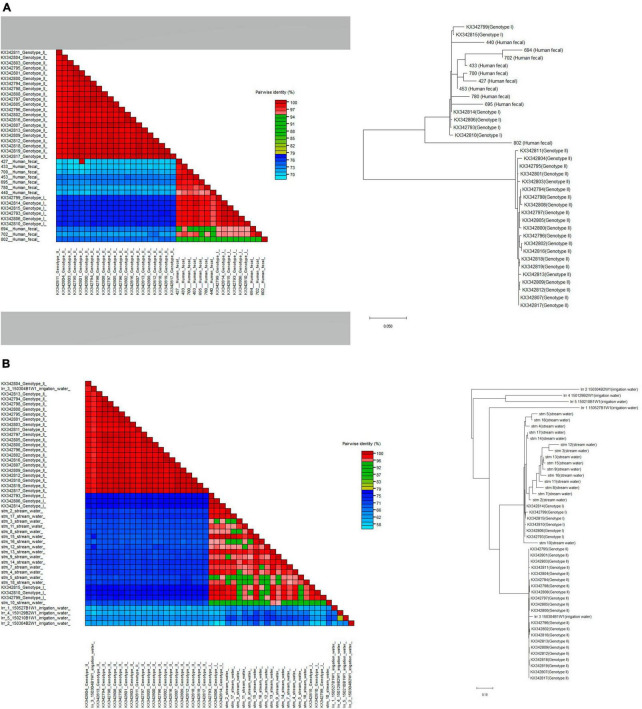
Phylogenetic relationships and pairwise sequence comparison of crAssphage strains from samples of **(A)** human feces and **(B)** water (stream and irrigation water). Color-coded pairwise identity matrix generated by Sequence Demarcation Tool (SDT v.1.2.) is located on the left. Each cell includes the percentage identity among 2 sequences (horizontally to the left and vertically at the bottom). The phylogenetic trees generated by the maximum-likelihood method were constructed using MEGA X and located on the right.

We successfully sequenced crAssphage from 10 human fecal specimens, 5 irrigation water samples, and 15 stream water samples. crAssphage sequences from human fecal samples and stream water samples could be typed as genotype I. In contrast, sequences from irrigation water samples formed a distinct cluster, which was separate from both genotypes I and II (Figure 4).

## 4 Discussion

The main goal of this study was to evaluate the usefulness of a novel human-associated phage, the crAssphage, as a pathogenic virus indicator for fresh leafy greens and environmental water contamination. We detected crAssphages not only in fresh leafy greens but also in stream water and irrigation water samples. Average detection rates of crAssphage ranged from 58.5% in stream water, which is comparable with previous reports in surface water (63 to 94%) (UK, Thailand, Japan, and Napal) (Farkas et al., 2019; Sabar et al., 2022). Related to fresh produce, analysis of three processing water samples obtained from baby leaves, bell peppers, and mixed veggie fruit processing facilities showed half (50%) of the washing (processing) water samples tested crAssphages positive (Cuevas-Ferrando et al., 2021). In support of this, our study demonstrated the average prevalence rates of crAssphages in irrigation water and fresh leafy greens were 61 and 68.5%, respectively. Our findings suggest that crAssphage could be used as markers to monitor fecal-borne virus contamination in key sources of fecal contamination in pre-harvest or post- harvest processes, offering potential improvements in food safety practices.

One of the primary challenges in detecting norovirus in environmental samples is their lower vial load. This contrasts with clinical matrices where viral loads often surpass 10^6^ infectious units/g of fecal materials (Lee et al., 2007; Atmar et al., 2008). In support of it, previous research and this study has indicated that the viral loads typically found in food and environmental samples rarely exceed 10^2^ infectious units per 25 gram (food) or 100 mL (water) (Mattison et al., 2010; Baert et al., 2011). Adding to the complexity, cell culture methods suitable for growing viruses, as is commonly done with bacteria, are not available for monitoring viral contamination in leafy greens. Consequently, additional technical steps to elute viruses from leafy greens and concentrate them are required to enhance viral detection (Croci et al., 2008). Unfortunately, these steps often lead to significant viral losses. We found that crAssphage were detected at 2-log higher concentration over norovirus. This agrees with reports from other viruses such as adenovirus, JC polyomavirus, and sapovirus (Stachler et al., 2017; Farkas et al., 2019; Malla et al., 2019). Thus, this suggests that crAssphage could be more readily detected than other fecal-borne viruses, thereby making it potentially a more sensitive indicator to monitor human fecal contamination.

Recent studies have demonstrated that crAssphage can be used to predict norovirus contamination in shellfish (Jennings et al., 2020; Gyawali et al., 2021). In addition, crAssphage was employed to monitor human fecal contamination on frequently touched surfaces on cruise ships and in long-term care facilities that experienced norovirus outbreaks, as well as to monitor the cleanliness of residents’ hands in these facilities (Park et al., 2020; Cannon et al., 2022). Our data show that while the presence of crAssphage does not always unequivocally indicate norovirus contamination, they can be used to identify contamination on frequently touched surfaces highlighting environmental surfaces that may require enhanced cleaning measures. We detected crAssphage in over 70% of norovirus-positive environmental samples suggesting that crAssphage can be a pivotal tool to assess potential health risks of exposure to contaminated water and food.

The two crAssphage assays (TN201-203 and CPQ056) we used in this study exhibited different sensitivities depending on the sample type. The TN201-203 assay demonstrated greater sensitivity for clinical samples, while the CPQ056 assay yielded more positive results with environmental samples. This sample-specific sensitivity suggests a possible benefit of combined use of both assays to more accurately monitor crAssphage in various environments. The CPQ056 assay targets the ORF00024 region, which encodes a hypothetical protein of crAssphage genome (JQ995537) (Stachler et al., 2017). In contrast, the TN201-203 target conserved regions of the DNA polymerase gene (ORF00018) found in a range of crAssphage sequences (Park et al., 2020). However, differences in detection rates of the two assays may also be caused by the presence of animal fecal matter in environmental samples that contain crAssphage (Stachler et al., 2017; Malla et al., 2019). Therefore, further refinement of the crAssphage assay may be required to make the assay 100% specific for human feces contaminated samples.

In alignment with prior research, our findings corroborate the global geographic distribution of crAssphage, though we note variations in its prevalence. These discrepancies can be attributed to several factors. Firstly, dietary patterns seem to impact the prevalence of crAssphage in human populations, with higher rates found in communities consuming meat-based diets. The high-fiber but relatively low-meat dietary pattern prevalent in South Korea might account for the lower prevalence rate of crAssphage observed in our study (Edwards et al., 2019; Honap et al., 2020). Secondly, the PCR assays used in our research, while well-validated and commonly employed, are designed based on only one of the ten recognized genera of crAssphage. This specificity, along with the genetic diversity and geographical variations of crAssphage, could limit the scope of detection. Despite these challenges, it is crucial to acknowledge that water sources—key conduits for contaminating leafy greens—represent combined contamination from multiple infected individuals, rather than from a single source. Thus, we assert that crAssphage can be an effective indicator for assessing viral contamination in such environments.

Our study has several limitations. First, the leafy green samples tested in this study were selected from limited geographical areas in South Korea and therefore it is unclear if our findings can be generalized. Second, in addition to the possible detection of crAssphage in non-human samples, the two crAssphage assays were both validated against only one of the 10 reported crAssphage genera (Stachler et al., 2017; Park et al., 2020). While the PCR assays used in our research are well-validated and have seen application in other studies, it’s important to note that these assays were developed based on a single genus out of the ten recognized genera of crAssphage. Given the genetic diversity of crAssphage and its geographical variations, there is a clear need for further refinement of these assays. Third, the detection of viruses through PCR techniques, as employed in this study, does not necessarily indicate infectivity. Recognizing that PCR-based detection methods may not directly translate to health risks associated with viral infections adds a layer of complexity to the interpretation of our results. Further studies exploring the infectivity of the detected viruses would contribute valuable insights into the actual health implications of viral presence in the analyzed samples. Last, given the widespread use of pepper mild mottle virus (PMMV) as a fecal indicator (Rosario et al., 2009), it is imperative for future research to include a comparative analysis with crAssphage to assess their relative effectiveness as indicators.

In conclusion, crAssphages were frequently detected in a variety of environmental water sources including irrigation and stream water. Notably, a significant correlation was observed between the presence of crAssphage and human norovirus contamination in fresh leafy greens. Furthermore, the concentration of crAssphage in the tested samples was consistently found to be at least 2 log10 units higher than that of norovirus, with over 70% of norovirus-positive samples also contaminated with crAssphage. These findings suggest that crAssphages may serve as reliable indicators of fecal-borne virus contamination and potentially of foodborne viruses, such as norovirus and hepatitis A virus. The application of crAssphage detection as biological markers could significantly enhance the safety of food and water supplies. Additional studies are warranted to explore the potential use of crAssphage as a pre-harvest biomarker to monitor fecal contamination in other fresh produce.

## Data availability statement

The datasets presented in this study can be found in online repositories. The names of the repository/repositories and accession number(s) can be found in this article/supplementary material.

## Author contributions

SS: Formal Analysis, Investigation, Methodology, Writing – original draft, Writing – review & editing. JL: Conceptualization, Investigation, Methodology, Project administration, Writing – review & editing. SeK: Methodology, Project administration, Resources, Validation, Writing – review & editing. JV: Conceptualization, Methodology, Resources, Validation, Writing – review & editing. SoK: Conceptualization, Funding acquisition, Project administration, Resources, Supervision, Writing – review & editing. GP: Conceptualization, Formal Analysis, Methodology, Validation, Visualization, Writing – original draft, Writing – review & editing.
